# Correction: Dynamic Advisor-Based Ensemble (dynABE): Case study in stock trend prediction of critical metal companies

**DOI:** 10.1371/journal.pone.0214339

**Published:** 2019-03-19

**Authors:** Zhengyang Dong

The values in Table 8 were calculated incorrectly. Please see the corrected Table 8 here.

**Table 8:** Evaluations on trading strategies. The returns have been annualized using 250 days as the number of trading days in a year.

Company	Annualized Absolute Return (%)	Annualized Excess Return to Stock (%)	Annualized Excess Return to Index (%)	Sharpe Ratio	Maximum Drawdown (%)
Jinchuan	184.97	90.40	165.95	1.51	24.79
Sumitomo	262.47	246.57	244.33	1.57	9.60
Zijin	71.10	69.88	48.65	1.99	10.68

In the Abstract, there is an error in the penultimate sentence of the first paragraph. The correct sentence is: We test dynABE on three cobalt-related companies, and it achieves the best-case misclassification error of 31.12% and an annualized absolute return of 262.47% with 9.60% maximum drawdown.

In the 5.6 Trading strategy performance subsection of the Results and analysis, there is an error in the last sentence of the third paragraph. The correct sentence is: We see that the trading strategy on Jinchuan has a maximum drawdown of 24.79%, Sumitomo 9.60%, and Zijin 10.68%.

In the Conclusion, there is an error in the first sentence of the first paragraph. The correct sentence is: By achieving a best-case misclassification error of 31.12% for Jinchuan Group and a best-case profit of 262.47% annualized absolute return for Sumitomo Metal Mining, dynABE demonstrates accurate stock predictions and high profitability.
