# Supplementary material for: Dynamic Advisor-Based Ensemble (dynABE): Case study in stock trend prediction of critical metal companies
Source: PLoS One. 2019 Feb 22;14(2):e0212487. doi: 10.1371/journal.pone.0212487 (PMC6386270; doi:10.1371/journal.pone.0212487)
Supplement: S1 Appendix — We provide the details of preprocessing and cleaning the data. (PDF) [file pone.0212487.s001.pdf]

S1 Appendix. Preprocessing procedures.

Here we introduce the preprocessing procedures for the data before it goes into feature selection. All data is cleaned at first. Since the purpose of this research is to predict the stock trend on a *daily* basis, less dynamic features with more than 50% of the observation being static are omitted. Afterward, all NA values in the features are replaced with the last non-NA observation. In doing so, the difference from the last non-NA observation will be reflected in the next non-NA observation, ignoring the NA observations in between.

After the cleaning, all observations are first-differenced in order to satisfy the stationarity assumptions of regression models. We have conducted an Augmented Dickey-Fuller (ADF) test for stationarity analysis on our feature set, and the result of which is included in S3 File. We observe that most of the original features are not stable, with ADF test p-values greater than 0.5, but all the features after first-differencing are stable, with ADF test p-values all smaller than 0.01. Although there is a small portion of features that are already stable before first-differencing, we first-difference the entire feature set anyways for consistency. After first-differencing, we take the first five lags of all the features, which quintuples the number of features. This is because we can only use past data for future predictions, and a total of five lags can roughly capture information in the period of a week prior to the date of prediction.

Furthermore, highly correlated features within the feature set are omitted in order to reduce feature redundancies. Specifically, we make a correlation matrix and remove one feature in the pair of features whose correlation is higher than 0.95. Fig 1 shows an example correlation plot for a subset of features. We display the specific correlation coefficient values in the plot. Moreover, we also calculate the p-values of the correlations, and those with p-values greater than 0.01 are displayed with a cross over them.

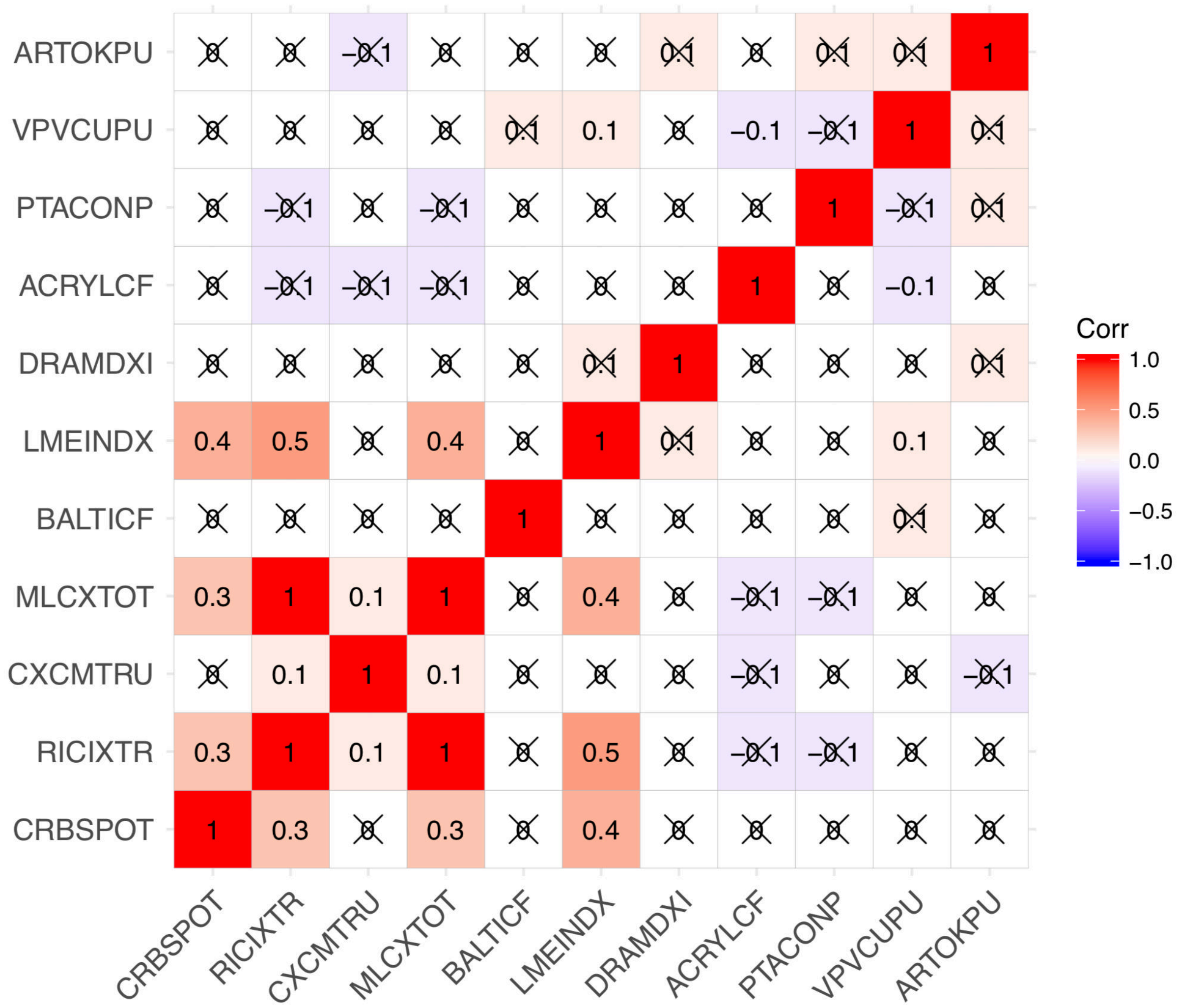

Fig 1. Correlation plot for a subset of features.
Values of the coefficient of correlation are labeled. Correlations with p-values smaller than 0.01 are crossed out. Features are displayed as their symbols, corresponding to descriptions in S2 File.

Finally, before feature selection, the stock trend is converted from continuous, first-differenced values to binary labels. Values greater than or equal to 0 are converted to 1 to represent a rise in price, and negative values are converted to 0 to represent a fall in price.
